# Vulnerability to unhealthy behaviours across different age groups in Swedish Adolescents: a cross-sectional study

**DOI:** 10.1080/21642850.2014.892429

**Published:** 2014-03-10

**Authors:** Ulrica Paulsson Do, Birgitta Edlund, Christina Stenhammar, Ragnar Westerling

**Affiliations:** ^a^ Department of Public Health and Caring Sciences, Uppsala University, Uppsala, Sweden

**Keywords:** adolescents, vulnerability, health-related behaviour, sociodemographic position, age factors

## Abstract

*Purpose*: There is lack of evidence on the effects of health-promoting programmes among adolescents. Health behaviour models and studies seldom compare the underlying factors of unhealthy behaviours between different adolescent age groups. The main objective of this study was to investigate factors including sociodemographic parameters that were associated with vulnerability to health-damaging behaviours and non-adoption of health-enhancing behaviours in different adolescent age groups. *Methods*: A survey was conducted among 10,590 pupils in the age groups of 13–14, 15–16 and 17–18 years. Structural equation modelling was performed to determine whether health-damaging behaviours (smoking and alcohol consumption) and non-adoption of health-enhancing behaviours (regular meal habits and physical activity) shared an underlying vulnerability. This method was also used to determine whether gender and socio-economic status were associated with an underlying vulnerability to unhealthy behaviours. *Results*: The findings gave rise to three models, which may reflect the underlying vulnerability to health-damaging behaviours and non-adoption of health-enhancing behaviours at different ages during adolescence. The four behaviours shared what was interpreted as an underlying vulnerability in the 15–16-year-old age group. In the youngest group, all behaviours except for non-participation in physical activity shared an underlying vulnerability. Similarly, alcohol consumption did not form part of the underlying vulnerability in the oldest group. Lower socio-economic status was associated with an underlying vulnerability in all the age groups; female gender was associated with vulnerability in the youngest adolescents and male gender among the oldest adolescents. *Conclusions*: These results suggest that intervention studies should investigate the benefits of health-promoting programmes designed to prevent health-damaging behaviours and promote health-enhancing behaviours in adolescents of different ages. Future studies should examine other factors that may contribute to the underlying vulnerability in different age groups.

## 1. Background

Adolescence is a period of life when many health-related behaviours, including smoking, alcohol consumption, regular meal habits and level of physical activity, become set for later years (Hallal, Victora, Azevedo, & Wells, 2006; Kelder, Perry, Klepp, & Lytle, 1994; Paavola, Vartiainen, & Haukkala, 2004).

Lower socio-economic status (Abudayya, Stigum, Shi, Abed, & Holmboe-Ottesen, 2009; Hanson & Chen, 2007a; Hoglund, Samuelson, & Mark, 1998; Neumark-Sztainer et al., 1996; Seabra, Mendonca, Thomis, Anjos, & Maia, 2008) and female gender (Epstein et al., 2001; Galanti, Rosendahl, Post, & Gilljam, 2001; Seabra et al., 2008; Trost et al., 2002) have been found to be associated with unhealthy behaviours among adolescents. The presence of unhealthy behaviours also increase with age during adolescence (Flay, 2002; Kahn et al., 2008; Neumark-Sztainer et al., 1996; van Nieuwenhuijzen et al., 2009; Seabra et al., 2008; Trost et al., 2002). Variations have been found for different unhealthy behaviours, such as smoking, alcohol consumption, lower levels of physical activity, irregular meal habits and poor nutritional intake.

Health behaviour studies that investigate one underlying factor are far more common than those investigating multiple factors (Peters et al., 2009). However, it is evident in the literature that underlying factors of unhealthy behaviours often occur in clusters (Peters et al., 2009; Trost et al., 2002). This clustering may be interpreted as an underlying vulnerability to unhealthy behaviours (Blum & Blum, 2009; Donovan & Jessor, 1985; Donovan, Jessor, & Costa, 1988; Shi & Stevens, 2010).

The implementation of health-promoting programmes may be a good strategy for encouraging adolescents with an underlying vulnerability to adopt healthy behaviours. Despite decades of health-promoting initiatives (Allen, Hauser, Bell, & O'Connor, 1994; Thompson, 1978), however, the effects of health programmes have often been limited (Thompson, 1978; Van Cauwenberghe et al., 2010; Wiehe, Garrison, Christakis, Ebel, & Rivara, 2005) or unclear (Mukoma & Flisher, 2004; St Leger, 1999). To be effective, health-promoting programmes should be formulated using scientifically based data.

Health-related behaviours are often studied in isolation from one another. However, Aaro, Laberg, and Wold (1995) studied the clustering of adolescent health-related behaviours and presented the idea of there being two dimensions. One dimension was health-damaging behaviours (such as smoking and alcohol consumption) and the other was health-enhancing behaviours (such as regular meal habits and physical activity). They called this the Hypothesis of Two Dimensions (Aaro et al., 1995). Researchers have argued that health programmes for adolescents could benefit from further development of the health behaviour model (DiClemente, Santelli, & Crosby, 2009; Langer & Warheit, 1992). This hypothesis may explain the low number of studies and lack of scientifically based models investigating these two types of behaviours together (Aaro et al., 1995; Giannakopoulos, Panagiotakos, Mihas, & Tountas, 2009; Kulbok & Cox, 2002; Peters et al., 2009).

The Model of Resilience in Adolescence reflects a vulnerability resulting from a convergence of many underlying factors that may affect health-related behaviours in general (Blum & Blum, 2009). However, Jessor and colleagues initiated studies into the underlying vulnerability to a set of specific health-damaging behaviours among adolescents (Donovan & Jessor, 1985; Donovan et al., 1988; Jessor & Jessor, 1977). In their investigations, performed in the USA, they identified several underlying factors, which were interpreted as a form of vulnerability to problem behaviours (Donovan & Jessor, 1985; Donovan et al., 1988; Turbin, Jessor, & Costa, 2000). These studies laid the basis for Problem Behaviour Theory (Donovan & Jessor, 1985; Jessor & Jessor, 1977). However, scientifically based structures of an underlying vulnerability, comprising factors that affect also non-adoption of health-enhancing behaviours, have not been well studied.

Similarly, no such age-specific health behaviour models have been targeted at adolescents. As argued by Langer and Warheit (1992), this can be problematic because underlying factors of health-related behaviours may vary between age groups (Galanti et al., 2001; van Nieuwenhuijzen et al., 2009; Seabra et al., 2008; Trost et al., 2002). Differences in specific unhealthy behaviours have previously been found between age groups during adolescence (Flay, 2002; Kahn et al., 2008; Neumark-Sztainer et al., 1996; van Nieuwenhuijzen et al., 2009; Seabra et al., 2008; Trost et al., 2002). However, there is a need for analysing common vulnerability factors for unhealthy behaviours among different age groups. Findings in this area could improve our understanding towards designing health-promoting programmes for adolescents that are more effective.

**Figure 1.**
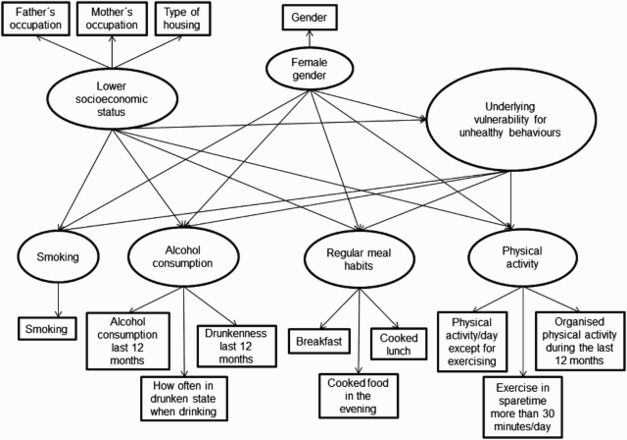
Hypothesised path model. A hypothesised path model of an underlying vulnerability to health-damaging behaviours (smoking and alcohol consumption) and non-participation in health-enhancing behaviours (regular meal habits and physical activity) in adolescents aged 13–18 years. The rectangular boxes represent indicator variables for the first-order latent variables. The small ovals represent first-order latent variables and the large oval represents a second-order latent variable.

The objective of the current study was to determine whether there is a shared underlying vulnerability to health-damaging behaviours (smoking and alcohol consumption) and non-adoption of health-enhancing behaviours (regular meal habits and physical activity) in different age groups during adolescence. It was hypothesised (Figure 1) that these four behaviours share an underlying vulnerability in all age groups. Another objective was to determine the extent to which socio-economic status and gender contributed to this hypothesised underlying vulnerability in these age groups. Hereinafter in this article, ‘unhealthy behaviours’ refers to health-damaging behaviours (smoking and alcohol consumption) and non-adoption of health-enhancing behaviours (regular meal habits and physical activity).

## 2. Methods

### 2.1. Sample

Of the 12,312 adolescents who were invited to take part in this study, 10,590 (86%) actually participated. They included 3664 of 4071 adolescents aged 13–14 years (90%), 4025 of 4522 adolescents aged 15–16 years (89%) and 2901 of 3719 adolescents aged 17–18 years (78%).

### 2.2. Procedure

A self-reported questionnaire about health, living habits and the essentials of life called ‘Life and Health – Young People 2007’ (*Liv och Hälsa – Ung*) was distributed to pupils in school grades seven (13–14-year-olds) and nine (15–16-year-olds) in upper primary school and in school grade two in upper secondary school (17–18-year-olds). Teachers distributed the questionnaires to the adolescents during ordinary classes in May 2007. The pupils were informed about the aims and confidentiality of the study on the first page of the questionnaire. Participation was voluntary and the adolescents who answered the questionnaire gave their informed consent. There were non-responses as a result of absence through illness on the day of the questionnaire being handed out.

The questionnaire consisted of 86 items for adolescents aged 13–14 years and 136 items for those aged 15–18 years. Since 1995, this questionnaire has been distributed every second year to adolescents by several counties in Sweden and scientific analyses based upon the results have been published (Brunnberg, Bostrom, & Berglund, 2008; Brunnberg, Linden-Bostrom, & Berglund, 2008). The items in the questionnaire were based on the Alcohol Use Disorders Identification Test (Bergman, Kallmen, Rydberg, & Sandahl, 1998; Reinert & Allen, 2007; Saunders, Aasland, Babor, de la Fuente, & Grant, 1993), the Annual National Study of Alcohol and Drug Habits of School Children (Andersson, Hansagi, Damstrom Thakker, & Hibell, 2002), Survey on Living Conditions (Jonsson & Östberg, 2010) and Health on Equal Terms (‘Health on equal terms – national goals for public health’, 2001). Fifteen questions were used in the present study (Table 1); these have also been used in other published studies (Andersson et al., 2002; Brunnberg, Bostrom, et al., 2008; Brunnberg, Linden-Bostrom, et al., 2008; Telama et al., 2005).

**Table 1.** Variables included in the analyses and distribution of answers.

First-order latent variables	Indicating variables for first-order latent variables (number of respondents)	Answer alternatives	Frequency (%)
Socio-economic status	Father's occupation (10,395)	Unemployed or long-term sick-listed	6.3
		Study, parental leave/house-husband or other activity	4.9
		Employed	88.8
	Mother's occupation (10,483)	Unemployed or long-term sick-listed	8.8
		Study, parental leave/housewife or other activity	9.5
		Employed	81.7
	Type of housing (10,479)	Leasehold flat	13.5
		Cooperative or time-share apartment	9.2
		Townhouse, semi-detached house or villa	77.3
Gender	Gender (10,519)	Boys	50.0
		Girls	50.0
Smoking	Smoking (10,422)	No (I have never smoked, I have tried, I have stopped)	81.4
		Yes (I smoke occasionally or daily)	18.6
		Alcohol consumption	Alcohol consumption last 12 months (10,282)^a^	Never	48.2
				Less than every second month – about once/month	33.6
				Twice/month – more than 4 times/week	18.2
	Drunkenness last 12 months^b^ (5706)		Never or in a sporadic manner	31.1	
			Some or a few times per year	26.0	
			Once/month	17.9	
			Twice/month – daily	25.0	
	How often in drunken state when drinking^b^ (5677)		Never/seldom	38.0	
			Occasionally	18.2	
			Almost every time–every time	43.8	
Regular meal habits	How often do you eat the following meals during a normal week?				
	Breakfast (10,410)	Seldom/never	8.2		
		1–3 days	8.4		
		4–6 days	15.1		
		Every day	68.3		
	Cooked lunch (10,383)	Seldom/never	3.4		
		1–3 days	7.7		
		4–6 days	27.5		
		Every day	61.4		
	Cooked food in the evening (10,391)	Seldom/never	2.0		
		1–3 days	3.9		
		4–6 days	13.7		
		Every day	80.4		
Physical activity	Physical activity/day except for exercising (10,259)	Less than 15 min	10.3		
		15–30 min	36.2		
		31–60 min	28.3		
		More than 1 hour	25.2		
	Exercise in spare time more than 30 minutes/day (10,333)	0–3 times/month	20.6		
		1–3 times/week	46.0		
		4 times/week – every day	33.3		
	Organised physical activity during the last 12 months (10,376)	No	29.1		
		Yes	70.9		

^a^Response alternatives were ‘never’, ‘every second month or less than every second month’, ‘about once per month’, ‘two to four times/per month’, ‘two to three times per week’ and ‘four times per week or more’ for adolescents aged 15–18 years and ‘never’, ‘every second month or less than every second month’, ‘about once per month’ and ‘twice per month or more often’ for adolescents aged 13–14 years.

^b^Question was asked of adolescents aged 15–16 years and 17–18 years.

The present study started in 2003 and followed the ethical guidelines for humanistic and social science research in Sweden (Law, 2003:460). The study was performed in accordance with the Declaration of Helsinki and the ethical standards of the ethics committee at Uppsala University, Sweden. Following Swedish law (Law, 2003:460), the study was granted exemption from requiring ethical approval as stated by the ethical committee at Uppsala University (which determined that ethical approval was not required).

### 2.3. Study variables

Measurement modelling analysis, correlation analysis and structural equation modelling (SEM) analysis were performed using the statistical program LISREL 8.80 (Jöreskog & Sörbom, 1993). Pairwise deletion was employed to deal with missing values.

#### 2.3.1. Variables

Questions used in this study related to sociodemographic variables, health-damaging behaviour variables and health-enhancing behaviour variables (Table 1). The questionnaire included two items relating to sociodemographic variables – ‘gender’ and ‘school grade’. The items ‘housing’, ‘occupational status of the mother’ and ‘occupational status of the father’ are recommended socio-economic measures for children (Hauser, 1994) and they were used as indicators of socio-economic status. The health-damaging behaviour variables included in this study related to ‘smoking’ and ‘alcohol consumption’ and the health-enhancing behaviour variables related to ‘regularity of meal habits’ and ‘physical activity’.

#### 2.3.2. Latent variables

The variables in this study were measured in LISREL as first-order latent variables (i.e. constructs of one or a number of indicating variables), which were hypothesised as representing latent phenomena (Diamantopoulos & Siguaw, 2007), such as ‘socio-economic status’. The validity and reliability of these variables were obtained employing measurement modelling analysis (Diamantopoulos & Siguaw, 2007). These analyses were performed (one analysis for each age group; Table 2) to ensure that the indicator variables chosen for each first-order latent variable were significantly loaded onto their intended latent variables (as indicated by path coefficients) and could be included as latent variables in the correlation analyses and in the SEM analyses in LISREL.

**Table 2.** Path coefficients in measurement modelling analyses.

Age group^a^ (*N*)	First-order latent variable	Indicator variables	Path coefficient (95% CI)
13–14 years (3664)	Smoking	Smoking	1.00^b^
	Alcohol consumption	Alcohol consumption	1.00^b^
	Regular meal habits	Breakfast	0.67 (0.63–0.71)***
		Lunch	0.63 (0.59–0.67)***
		Evening meal	0.60 (0.56–0.64)***
	Physical activity	Physical activity per day	0.28 (0.23–0.33)***
		Exercise in spare time	0.58 (0.53–0.63)***
		Organised physical activity	0.73 (0.67–0.79)***
	Socio-economic status	Father's occupation	0.68 (0.64–0.72)***
		Mother's occupation	0.46 (0.42–0.50)***
		Housing	0.52 (0.48–0.56)***
	Gender	Gender	1.00^b^
15–16 years (4025)	Smoking	Smoking	1.00^b^
	Alcohol consumption	Alcohol consumption	0.87 (0.85–0.89)***
		Drunkenness	0.87 (0.85–0.89)***
		Drunken state	0.89 (0.77–0.93)***
	Regular meal habits	Breakfast	0.87 (0.84–0.90)***
		Lunch	0.74 (0.69–0.79)***
		Evening meal	0.60 (0.55–0.65)***
	Physical activity	Physical activity per day	0.11 (0.01–0.21)***
		Exercise in spare time	0.66 (0.60–0.72)***
		Organised physical activity	0.80 (0.74–0.86)***
	Socio-economic status	Father's occupation	0.69 (0.15–1.23)***
		Mother's occupation	0.65 (0.09–1.21)***
		Housing	0.43 (0.03–0.83)***
	Gender	Gender	1.00^b^
17–18 years (2901)	Smoking	Smoking	1.00^b^
	Alcohol consumption	Alcohol consumption	0.95 (0.92–0.98)***
		Drunkenness	0.95 (0.92–0.98)***
		Drunken state	0.69 (0.65–0.73)***
	Regular meal habits	Breakfast	0.57 (0.54–0.60)***
		Lunch	0.42 (0.36–0.48)***
		Evening meal	0.55 (0.49–0.61)***
	Physical activity	Physical activity per day	0.35 (0.31–0.39)***
		Exercise in spare time	0.88 (0.82–0.94)***
		Organised physical activity	0.57 (0.53–0.61)***
	Socio-economic status	Father's occupation	0.69 (0.64–0.74)***
		Mother's occupation	0.58 (0.47–0.63)***
		Housing	0.53 (0.51–0.55)***
	Gender	Gender	1.00^b^

Notes: Significance testing of how well indicator variables load with first-order latent variables. *Fit statistics*: 13–14 years: chi-square of 75.76 with 25 df, RMSEA of 0.02, GFI of 1.00, AGFI of 0.99 and RMR of 0.02. 15–16 years: chi-square of 44.08 with 21 df, RMSEA of 0.02, GFI of 1.00, AGFI of 0.99 and RMR of 0.01. 17–18 years: chi-square of 75.18 with 35 df, RMSEA of 0.02, GFI of 1.00, AGFI of 0.99 and RMR of 0.02.

CI, confidence interval.

^a^Measurement model analyses were performed for all adolescents in the data set as well as for each age group separately.

^b^When there is only one indicator variable for a first-order latent variable, the path coefficient becomes 1.00 and the 95% CI cannot be measured.

***Statistically significant at the 95% CI.

A second-order latent variable, which is a construct of a number of first-order latent variables (Kline, 2011; Schumacker, 2010), was used to test the hypothesis that adolescents with health-damaging behaviours (smoking or alcohol consumption) or non-adoption of health-enhancing behaviours (irregular meal habits or a low level of physical activity) share a common underlying vulnerability (Figure 1). A second-order latent variable was therefore measured through the indicative variables of the first-order latent variables such ‘smoking’, ‘alcohol consumption’, ‘regularity of meal habits’ and ‘physical activity’. Polychoric correlation analyses were performed in LISREL (one for each age group) (Table 3) to ensure that the second-order latent variables were significantly loaded onto these first-order latent variables (as indicated by correlation coefficients) and could be included as a second-order latent variable in the SEM analyses and correlation analysis in LISREL. The second-order latent variable in the SEM analyses was interpreted as an underlying vulnerability.

**Table 3.** Correlation coefficients of health behavioural variables and the underlying vulnerability.

Age group	1.	2.	3.	4.	5.	6.	7.
*13–14 years*							
1. Regular meal habits^a^	1.00*						
2. Physical activity^a^	0.18*	1.00*					
3. Smoking^a^	−0.59*	−0.15*	1.00*				
4. Alcohol consumption^a^	−0.51*	−0.08*	0.71*	1.00*			
5. Gender^a,b^	−0.27*	0.07*	0.06*	0.03*	1.00*		
6. High socio-economic status^a^	0.50*	0.42*	−0.36*	−0.20*	−0.06*	1.00*	
7. Underlying vulnerability^c^	−0.72*	−0.16*	0.82*	0.74*	0.41*	−0.46*	1.00*
*15–16 years*							
1. Regular meal habits^a^	1.00*						
2. Physical activity^a^	0.32*	1.00*					
3. Smoking^a^	−0.40*	−0.20*	1.00*				
4. Alcohol consumption^a^	−0.36*	−0.14*	0.77*	1.00*			
5. Gender^a,b^	−0.85*	−0.20*	0.02	0.09*	1.00*		
6. High socio-economic status^a^	0.41*	0.36*	−0.27*	−0.11*	−0.13*	1.00*	
7. Underlying vulnerability^c^	−0.36*	−0.16*	0.97*	0.79*	0.01	−0.13*	1.00*
*17–18 years*							
1. Regular meal habits^a^	1.00*						
2. Physical activity^a^	0.33*	1.00*					
3. Smoking^a^	−0.29*	−0.21*	1.00*				
4. Alcohol consumption^a^	−0.11*	−0.08*	0.55*	1.00*			
5. Gender^a,b^	−0.30*	−0.18*	−0.54*	−0.49*	1.00*		
6. High socio-economic status^a^	0.15*	0.24*	−0.12*	−0.05*	−0.13*	1.00*	
7. Underlying vulnerability^c^	−0.33*	−0.23*	0.90*	0.62*	−0.61*	−0.13*	1.00*

Notes: Polychoric correlation was used with a standardised solution (the standard deviation was set to 1 and the mean of all correlation coefficients was zero). *Fit statistics*: 13–14 years: chi-square of 100.02 with 22 df, RMSEA of 0.03, GFI of 1.00, AGFI of 0.98 and RMR of 0.02. 15–16 years: chi-square of 46.35 with 24 df, RMSEA of 0.02, GFI of 1.00, AGFI of 0.99 and RMR of 0.01. 17–18 years: chi-square of 120.57 with 42 df, RMSEA of 0.03, GFI of 0.99, AGFI of 0.99 and RMR of 0.02.

^a^First-order latent variable.

^b^Females vs. males.

^c^Second-order latent variable (which was included in the study to investigate a hypothesised underlying factor of unhealthy behaviours, referred to as the underlying vulnerability to unhealthy behaviours).

*Statistically significant (*p* < .05).

### 2.4. Statistical analyses

To determine whether age was associated with the underlying vulnerability, a polychoric correlation analysis was performed in LISREL, in which we investigated whether the first-order latent variable ‘age group’ would correlate with the underlying vulnerability.

A unit of measurement was then specified for the underlying vulnerability, i.e. the unstandardised direct effect of the underlying vulnerability was fixed at 1.00 (Kline, 2011). This is necessary for inclusion in the SEM analysis. In the correlation analyses (Table 3), ‘Smoking’ had the strongest loadings with the underlying vulnerability and it was therefore employed as the reference variable in the SEM analyses (Schumacker, 2010).

SEM analyses were performed separately for each age group. It was determined whether the health-damaging behaviours (‘smoking’ and ‘alcohol consumption’) and health-enhancing behaviours (‘regular meal habits’ and ‘physical activity’) reflected an underlying vulnerability for both health-damaging behaviours and non-adoption of health-enhancing behaviours (which we would interpret as a common underlying vulnerability to those behaviours). Using SEM, we also determined whether ‘socio-economic status’ and ‘gender’ were associated with the underlying vulnerability as well as with ‘smoking’, ‘alcohol consumption’, ‘regularity of meal habits’ and ‘physical activity’ in each age group. This analysis was performed to determine whether ‘socio-economic status’ and ‘gender’ were part of the possible underlying vulnerability to unhealthy behaviours that mediating these factors of both health-damaging behaviours and non-adoption of health-enhancing behaviours or whether low socio-economic status and gender were directly associated with individual unhealthy behaviours. The strengths of the associations were indicated by path coefficients. Owing to their low correlations (in the correlation analysis; Table 3), it was not possible to include some of the lowest correlations as paths in the SEM analysis such that the analysis could converge.

Path coefficients in the measurement modelling analyses and SEM analyses were assessed using confidence intervals, whereas the correlation analyses used the *p*-value. Model-fit measures, which present the level of conformity between the observation data and the models, were used to assess the correlation analyses, measurement model analyses and SEM analyses. The measures of fit used were chi-square, which should be non-significant (Schumacker, 2010); (numbers of) degrees of freedom (df); root mean square error of approximation (RMSEA, which should be below 0.08 to indicate an adequate fit); goodness-of-fit (GFI) and GFI index adjusted for degrees of freedom (AGFI), both of which should be above 0.90 for a good fit; and root mean square residual (RMR, which should be below 0.05) (Jöreskog & Sörbom, 1993; Schumacker, 2010).

## 3. Results

### 3.1. Descriptive analysis

Table 1 gives the distribution of adolescents in the study with regard to age group, socio-economic status and gender. The distribution of the adolescents’ health-related behaviours is also given in the table.

The measurement modelling analyses in LISREL 8.8 confirmed that the indicator variables for the different unhealthy behaviours in the study were significantly loaded onto the specific latent variables. The measurement modelling analyses also confirmed that the indicator variables for the latent variables were valid and reliable. The fit statistics, which assessed the plausibility of the measurement modelling analyses, indicated a good fit of the data in the analysis in all age groups (Table 2).

### 3.2. Correlation analyses of latent variables

‘Smoking’, ‘alcohol consumption’, ‘irregular meal habits’ and ‘low level of physical activity’ correlated with the underlying vulnerability in all age groups in the correlation analysis (Table 3). In the three age groups, the correlation coefficients were from 0.82 to 0.97 for ‘smoking’, 0.62 to 0.79 for ‘alcohol consumption’, −0.33 to −0.72 for ‘regularity of meal habits’ and −0.16 to −0.23 for ‘physical activity’. The correlation analyses therefore confirmed that there was an underlying vulnerability in all age groups studied. The correlation analyses showed that ‘smoking’ had the strongest association with the underlying vulnerability in all the age groups, whereas a ‘low level of physical activity’ had the weakest association in all age groups. The fit statistics of these correlation analyses indicated a good fit of the data in all the age groups.

The correlation analysis between ‘age group’ and the underlying vulnerability showed a statistically significant (*p* < .05) correlation coefficient of 0.95 (standardised solution). The fit statistics of this correlation analysis indicated a good fit of the data (chi-square of 61.58 with 16 df, RMSEA of 0.03, GFI of 1.00, AGFI of 0.98 and RMR of 0.02).

### 3.3. SEM analyses

**Figure 2.**
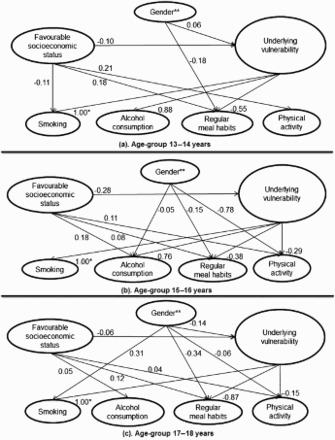
(a–c) Path coefficients of direct associations between a first-order and a second-order latent variable in three age groups.Notes: The path models (a–c) are SEM analyses (of different age groups) of the hypothesised model shown in Figure 1. To simplify the presentation, the indirect and total associations between the latent variables are omitted here, though they appear in Table 4. It should be noted that since the study is cross-sectional, the direction of causality is unknown. Associations are significant at the 95% CI. The small ovals represent first-order latent variables and the large ovals represent second-order latent variables. The second-order latent variable was interpreted as an underlying vulnerability to both health-damaging behaviours and non-participation in health-enhancing behaviours in all age groups.*To standardise the second-order latent variable, the path coefficient to the first-order latent variable ‘smoking’ was fixed at 1.00.**Females vs. males.

The path coefficients in the SEM analyses between the health-related behaviours and the underlying vulnerability in the three age groups were 1.00 for smoking in all groups; it was 0.88 for alcohol consumption in the youngest age group and 0.76 in the 15–16-year-old group. This path coefficient was non-significant in the oldest age group. The path coefficients between the underlying vulnerability and regularity of meal habits were ranged from −0.38 to −0.87 (which indicates a strong association with irregular meal habits). Engagement in physical activity, however, was only significantly associated with the underlying vulnerability in the two older age groups (−0.29 to −0.15), which indicates an association with a low level of physical activity (Table 4; Figure 2(a)–2(c)).

**Table 4.** Path coefficients of direct, indirect and total associations between underlying vulnerability and health behavioral variables in SEM analyses.

Age group	Direct association (95% CI)^a^	Indirect association (95% CI)^a^	Total association (95% CI)
13–14 years			
Underlying vulnerability:^b^			
Gender^c^	0.06 (0.04–0.08)*		0.06 (0.04–0.08)*
Higher socio-economic status	−0.10 (−0.12 to −0.08)*		−0.10 (−0.12 to −0.08)*
Regular meal habits:			
Second-order latent variable^b^	−0.55 (−0.57 to −0.53)*		−0.55 (−0.57 to −0.53)*
Gender^c^	−0.18 (−0.19 to −0.17)*	−0.03 (−0.04 to −0.02)*	−0.22 (−0.24 to −0.20)*
Higher socio-economic status	0.18 (0.16 to 0.20)*	0.06 (0.05 to 0.07)*	0.24 (0.22 to 0.26)*
Physical activity:			
Second-order latent variable^b^	−0.12 (−0.18 to −0.06)		−0.12 (−0.18 to −0.06)
Gender^c^		−0.01 (−0.01 to 0.01)*	−0.01 (−0.01 to −0.01)*
Higher socio-economic status	0.21 (0.12 to 0.30)*	0.01 (0.00 to 0.02)	0.22 (0.12 to 0.32)*
Smoking:			
Second-order latent variable^b^	1.00^d^		1.00^d^
Gender^c^		0.06 (0.04 to 0.08)*	0.06 (0.04 to 0.08)*
Higher socio-economic status	−0.11 (−0.12 to −0.10)*	−0.10 (−0.12 to −0.08)*	−0.21 (−0.23 to −0.19)*
Alcohol consumption			
Second-order latent variable^b^	0.88 (0.85 to 0.91)*		0.88 (0.85 to 0.91)*
Gender^c^		0.06 (0.05 to 0.07)*	0.06 (0.05 to 0.07)*
Higher socio-economic status		−0.09 (0.11 to 0.07)*	−0.09 (0.11 to 0.07)*
15–16 years			
Underlying vulnerability:^b^			
Gender^c^	−0.05 (−0.08 to −0.02)		−0.05 (−0.08 to −0.02)
Higher socio-economic status	−0.28 (−0.34 to −0.22)*		−0.28 (−0.34 to −0.22)*
Regular meal habits:			
Second-order latent variable^b^	−0.38 (−0.40 to −0.36)*		−0.38 (−0.40 to −0.36)*
Gender^c^	−0.15 (−0.17 to −0.13)*	0.02 (0.01 to 0.03)	−0.13 (−0.14 to −0.12)*
Higher socio-economic status	0.08 (0.05 to 0.11)*	0.11 (0.09 to 0.13)*	0.18 (0.17 to 0.19)*
Physical activity:			
Second-order latent variable^b^	−0.29 (−0.33 to −0.25)*		−0.29 (−0.33 to −0.25)*
Gender^c^	−0.78 (−0.89 to −0.67)*	0.02 (0.01 to 0.03)	−0.76 (−0.87 to 0.65)*
Higher socio-economic status	0.11 (0.08 to 0.14)*	0.08 (0.06 to 0.10)*	0.19 (0.17 to 0.21)*
Smoking:			
Second-order latent variable^b^	1.00^d^		1.00^d^
Gender^c^		−0.05 (−0.08 to −0.02)	−0.05 (−0.08 to −0.02)
Higher socio-economic status	0.11 (0.04 to −0.18)	−0.28 (−0.34 to −0.22)*	−0.18 (−0.19 to −0.17)*
Alcohol consumption:			
Second-order latent variable^b^	0.76 (0.71 to 0.81)*		0.76 (0.71 to 0.81)*
Gender^c^	−0.05 (−0.07 to −0.03)*	−0.04 (−0.06 to −0.02)	−0.09 (−0.11 to −0.07)*
Higher socio-economic status	0.18 (0.13–0.23)*	−0.21 (−0.26 to −0.16)*	−0.03 (−0.04 to −0.02)*
17–18 years			
Underlying vulnerability:^b^			
Gender^c^	−0.14 (−0.17 to −0.11)*		−0.14 (−0.17 to −0.11)*
Higher socio-economic status	−0.06 (−0.08 to −0.04)*		−0.06 (−0.08 to −0.04)*
Regular meal habits:			
Second-order latent variable^b^	−0.87 (−0.98 to −0.76)*		−0.87 (−0.98 to −0.76)*
Gender^c^	−0.34 (−0.37 to −0.31)*	0.12 (0.09–0.15)*	−0.22 (−0.24 to −0.20)*
Higher socio-economic status	0.12 (0.10–0.14)*	0.05 (0.03–0.07)*	0.17 (0.16–0.18)*
Physical activity:			
Second-order latent variable^b^	−0.15 (−0.18 to −0.12)*		−0.15 (−0.18 to −0.12)*
Gender^c^	−0.06 (−0.07 to −0.05)*	0.02 (0.01–0.03)	−0.04 (−0.05 to −0.03)*
Higher socio-economic status	0.04 (0.03–0.05)*	0.01 (0.01–0.01)*	0.05 (0.04–0.06)*
Smoking:			
Second-order latent variable^b^	1.00^d^		1.00^d^
Gender^c^	0.31 (0.28–0.34)*	−0.14 (−0.17 to −0.11)*	0.17 (0.15–0.19)*
Higher socio-economic status	0.04 (0.02–0.06)	−0.06 (−0.08 to −0.04)*	−0.10 (−0.11 to −0.09)*
Alcohol consumption:			
Second-order latent variable^b^	0.10 (0.03–0.17)		0.10 (0.03–0.17)
Gender^c^	0.01 (−0.01–0.03)	−0.01 (−0.02–0.00)	0.00 (−0.02–0.02)
Higher socio-economic status	0.05 (0.04–0.06)*	−0.01 (−0.01 to −0.01)*	−0.05 (−0.06 to −0.04)*

Notes: *Fit statistics*: 13–14 years: chi-square of 172.56 with 29 df, RMSEA of 0.04, GFI of 0.99, AGFI of 0.98 and RMR of 0.03. 15–16 years: chi-square of 153.24 with 37 df, RMSEA of 0.03, GFI of 0.99, AGFI of 0.98 and RMR of 0.02. 17–18 years: chi-square of 238.13 with 35 df, RMSEA of 0.05, GFI of 0.99, AGFI of 0.97 and RMR of 0.03.

*Statistically significant at the 95% CI.

^a^An empty cell indicates that no direct or indirect measurement was made between the two latent variables in question as an association between these was not included in the model that was tested.

^b^The second-order latent variable was interpreted as an underlying vulnerability for unhealthy behaviours. Remaining variables in the table are first-order latent variables.

^c^Females vs. males.

^d^To standardise the second-order latent variable, the path coefficient to the first-order latent variable ‘smoking’ was fixed to 1.00. Therefore, 95% CI could not be measured.

There was an association between female gender and unhealthy behaviours mediated by the underlying vulnerability in the youngest group (0.06) and there was an association with male gender in the oldest group (−0.14); the association in the 15–16-year-old group was non-significant (Table 4; Figure 2(a)–(2c)). The direct associations between gender and individual health-enhancing behaviours showed a connection with male gender in all the age groups, i.e. an association between female gender and non-participation in health-enhancing behaviours. Smoking (0.31) was also directly associated with female gender among the oldest adolescents, though the association with alcohol consumption was non-significant. When directly measured, the association between gender and unhealthy behaviours among the oldest adolescents thus differed from the direct measurement mediated by an underlying vulnerability (−0.14). However, the total associations showed connections with female gender (0.17).

There was an association between lower socio-economic status and unhealthy behaviours mediated by the underlying vulnerability in all the age groups (−0.06 to −0.28). Low socio-economic status and non-adoption of health-enhancing behaviours (i.e. regular meal habits and physical activity) were directly associated in all age groups. Direct associations that were tested and found to be significant between health-damaging behaviour (i.e. smoking and alcohol consumption) and socio-economic status showed a connection between smoking and low socio-economic status (−0.11) in the youngest group, i.e. the direct path showed the same association as the indirect association mediated by the underlying vulnerability (−0.10). In the two older age groups, however, the direct associations showed a connection between alcohol consumption and high socio-economic status and the indirect associations mediated by the underlying vulnerability showed a connection with low socio-economic status (−0.01 to −0.21). The direct and indirect associations thus differed. The total association between these variables, though, showed a low but significant association with low socio-economic status (−0.03 to −0.05).

The remaining indirect associations between gender and socio-economic status with health-related behaviour mediated by the underlying vulnerability appear under ‘Indirect association’ given in Table 4. Total associations (i.e. both direct and indirect associations) of gender and socio-economic status, for each health-related behaviour, are presented under ‘Total association’ given in Table 4.

The fit statistics, which assessed the plausibility of the SEM analyses, indicated a good fit of the data in all age groups.

## 4. Discussion

The purpose of this study was to assess whether health-damaging behaviours (smoking and alcohol consumption) and non-adoption of health-enhancing behaviours (regular meal habits and physical activity) share an underlying vulnerability that mediates these behaviours in three different age groups during adolescence. The second-order latent variable in the SEM analyses was interpreted as an underlying vulnerability. The underlying vulnerability was found to correlate strongly with age group during adolescence. We therefore chose to study the three age groups separately in the SEM analysis. The structural equation models for each age group demonstrated good levels of fit, which indicates that the sample data support the three models in the study. The GFI of the models had similar patterns and was therefore comparable.

The findings support the hypothesis about a common underlying vulnerability to both health-damaging behaviours and non-adoption of health-enhancing behaviours in all the age groups. These results may reflect the presence of an underlying vulnerability, which could be interpreted as a General Unhealthy Behaviour Vulnerability – in line with the General Model of Vulnerability (Shi & Stevens, 2010) and Model of Resilience in Adolescence (Blum & Blum, 2009). In those models, vulnerability reflects a convergence of multiple factors that affect a number of different areas of health (Shi & Stevens, 2010) or health-damaging behaviours and non-adoption of health-enhancing behaviours (Blum & Blum, 2009). The result of an underlying vulnerability in the present study has important implications for the design of health-promoting programmes for adolescents and indicates that multicomponent programming wherein the health-enhancing and preventive health-damaging activities are the goal should be considered.

The underlying vulnerability in the current study differed between the age groups: there was no association between the underlying vulnerability and level of physical activity in the youngest group and no association with alcohol consumption in the oldest group. Earlier investigations also found differences in unhealthy behaviours between age groups during adolescence (Flay, 2002; Kahn et al., 2008; Neumark-Sztainer et al., 1996; van Nieuwenhuijzen et al., 2009; Seabra et al., 2008; Trost et al., 2002). However, those studies investigated direct connections with unhealthy behaviours – not unhealthy behaviours mediated by an underlying vulnerability as in the present research. Kulbok and Cox (2002) studied multiple unhealthy behaviours in American adolescents and found that a low level of physical activity was only weakly associated with other unhealthy behaviours. This suggests that a low level of physical activity in young adolescents may be independent of other unhealthy behaviours in both the USA and Sweden. The finding of a vulnerability to irregular meal habits, low level of physical activity and smoking – but not to alcohol consumption – among the older adolescents was surprising; however, many studies have found a strong correlation between smoking and alcohol consumption (Karvonen, Abel, Calmonte, & Rimpela, 2000; Wiefferink et al., 2006). The findings of the present study lay a basis for longitudinal investigations of an underlying vulnerability to health-damaging behaviours and non-adoption of health-enhancing behaviours at different ages of adolescence.

In the present study, gender contributed to the underlying vulnerability in the youngest and oldest age groups. Although the associations were weak, we found girls to have an underlying vulnerability to both health-damaging behaviours and non-adoption of health-enhancing behaviours in early adolescence. In late adolescence, an underlying vulnerability was associated with boys in the current study; however, every single unhealthy behaviour was directly connected among girls. This indicates that boys are more vulnerable to multiple unhealthy behaviours, whereas girls are more directly connected with single unhealthy behaviours. For instance, irregular meal habits seem to be more common among girls in all ages and for girls in later adolescents (17–18 years) a lower level of physical activity seems to be a specific problem.

It is common knowledge that there are gender differences when it comes to unhealthy behaviours and it is also well known from earlier studies that girls more often have unhealthy behaviours than do boys (Abudayya et al., 2009; Mazur & Woynarowska, 2004; van Nieuwenhuijzen et al., 2009). Mazur and Woynarowska (2004) studied a cluster of unhealthy behaviours and found a relation with male gender. Their findings and the findings for the 17–18-year-old age group in the present study indicate a need for further investigations into multiple unhealthy behaviours and the underlying vulnerability to such behaviours. Our findings suggest that among older adolescents, girls' needs may be targeted by health-promoting programmes for individual unhealthy behaviours, such as a low level of exercise and irregular meal habits, whereas for girls in early adolescence and boys in late adolescence it might be better to address multiple unhealthy behaviours together, focusing on certain vulnerability factors associated with these behaviours.

In the case of mid-adolescence, a pattern similar to the ‘Hypothesis of Two Dimensions’ by Aaro et al. (1995) was found for the 15–16-year-old age group in the current study. Whereas girls showed a direct association with non-adoption of health-enhancing behaviours, boys evidenced a direct connection with alcohol consumption. Similarly, direct associations between unhealthy behaviours and socio-economic status followed different patterns for the two types of behaviour. Non-participation in health-enhancing behaviours was directly connected with low socio-economic status, whereas health-damaging behaviours were directly connected with high socio-economic status. These findings indicate that it may be appropriate to tackle health-damaging behaviours and non-adoption of health-enhancing behaviours separately in health-promoting programmes for 15–16-year-old adolescents. However, these behaviours could also be addressed together: an underlying vulnerability was found in this age group, whereby low socio-economic status was mediated by vulnerability to both non-adoption of health-enhancing behaviours and health-damaging behaviours. Further possible vulnerability factors need to be identified, in designing prevention efforts.

The health-damaging behaviours and non-adoption of health-enhancing behaviours were mediated by an underlying vulnerability among adolescents with a low socio-economic status among both the youngest and the oldest adolescents. However, the direct connections between the health-related behaviours and socio-economic status in the two older age groups showed, however, that alcohol consumption was directly connected with high socio-economic status. Since lower socio-economic status has been identified as being associated with isolated unhealthy behaviours (Hanson & Chen, 2007a), the direct associations with high socio-economic status in the two older age groups were surprising. However, substance use has previously been connected with high socio-economic status among adolescents (Hanson & Chen, 2007b). The higher level of alcohol consumption among the pupils from higher socio-economic groups may reflect a specific lifestyle pattern among these groups not connected to a general vulnerability.

Furthermore, a review study by Hanson and Chen (2007a) concluded that low socio-economic status is not as strongly associated with unhealthy behaviours in adolescents as in adults. This may explain the very low associations in some instances in the present study between socio-economic status and unhealthy behaviours. The two different patterns suggest that adolescents in the low socio-economic groups are vulnerable to unhealthy behaviours in general, whereas adolescents aged 15–18 years with a high socio-economic status are at risk of developing individual health-damaging behaviour. These findings highlight the importance of continued research into underlying vulnerability to unhealthy behaviours compared with studies of direct associations between unhealthy behaviours and socio-economic status. Our findings also support health-promoting programmes that address both health-damaging behaviours and non-adoption of health-enhancing behaviours among adolescents in low socio-economic groups. The higher socio-economic groups, on the other hand, may advantage from targeted programmes aiming at reducing alcohol consumption.

### 4.1. Strengths and limitations

The present study adds to several aspects of existing research. It is one of only a few investigations that have analysed common underlying factors of both health-damaging behaviours and non-adoption of health-enhancing behaviours. To our knowledge, it is the only study that includes smoking, alcohol consumption, regularity of meal habits and physical activity, combined with an analysis of a shared underlying vulnerability of clustering factors in different age groups during adolescence. Strong features of the study are also its unusually large sample and its design.

There were limitations to the study, however, which may have had an impact on the findings. The present study was cross-sectional, which prohibits prediction of future behaviours from the results. Furthermore, three questions measured alcohol consumption behaviour in the two oldest age groups, but only one of these questions (‘alcohol consumption in the last 12 months’) was used for the youngest group. There was also a slight difference among the groups in the number of response alternatives for this question. This makes comparisons between the age groups more difficult and it could have biased the comparisons among the age groups in the results and conclusions. However, it has been shown that among Swedish adolescents, alcohol consumption is slight for 13–14-year-olds, but increases substantially among 15–16-year-olds (Hvitfeldt & Rask, 2005). The relevance in asking the youngest group the two questions about alcohol consumption is therefore questionable. For SEM analyses to be useful, there has to be a variation in the answers given (Schumacker, 2010). Another limitation is that the study was undertaken in only one county. The geographical area of residence can affect health-related behaviours (Villard, Ryden, & Stahle, 2007). The large sample, however, allowed for the differences regarding socio-economic status, gender and age groups to appear.

### 4.2. Conclusions

The present study provides a novel insight into the vulnerability to health-damaging behaviours (smoking and alcohol consumption) and to non-adoption of health-enhancing behaviours (regular meal habits and physical activity) among adolescents. The findings indicate that during adolescence, vulnerability to these behaviours varies depending on age. Different behaviours may therefore be addressed together in different age groups. The results of this study do not offer a solution to the problems currently faced by health-promoting programmes for adolescents (DiClemente et al., 2009). However, intervention studies should investigate whether there is a benefit to jointly addressing health-damaging behaviours and non-adoption of health-enhancing behaviours in health-promoting programmes for 13–18-year-olds. Intervention studies should also investigate possible benefits of age-specific health-promoting programmes for adolescents. Finally, longitudinal studies should investigate further possible factors of vulnerability to health-damaging behaviours and non-adoption of health-enhancing behaviours in different age groups during adolescence.
