# Erratum

**DOI:** 10.1080/21642850.2014.916514

**Published:** 2014-05-30

Paulsson Do, U., Edlund, B., Stenhammar, C., & Westerling, R. (2014). Vulnerability to unhealthy behaviours across different age groups in Swedish Adolescents: a cross-sectional study. *Health Psychology and Behavioral Medicine*, *2*(1), 296–313. http://dx.doi.org/10.1080/21642850.2014.892429

When the above article was first published online, the following Acknowledgement Section was omitted in error. This has now been amended in the online version of this article.

